# Bell-shaped and ultrasensitive dose-response in phosphorylation-dephosphorylation cycles: the role of kinase-phosphatase complex formation

**DOI:** 10.1186/1752-0509-6-26

**Published:** 2012-04-24

**Authors:** Barbara Szomolay, Vahid Shahrezaei

**Affiliations:** 1 Department of Mathematics, Imperial College London, South Kensington Campus, SW7 2AZ London, UK; 2 Mathematics Institute Zeeman Building, University of Warwick, Coventry CV4 7AL, UK

**Keywords:** Cellular signalling, Zero-order ultrasensitivity, Phosphoinositide regulation, Endosomal trafficking

## Abstract

**Background:**

Phosphorylation-dephosphorylation cycles (PDCs) mediated by kinases and phosphatases are common in cellular signalling. Kinetic modelling of PDCs has shown that these systems can exhibit a variety of input-output (dose-response) behaviors including graded response, ultrasensitivity and bistability. In addition to proteins, there are a class of lipids known as phosphoinositides (PIs) that can be phosphorylated. Experimental studies have revealed the formation of an antagonistic kinase-phosphatase complex in regulation of phosphorylation of PIs. However, the functional significance of this type of complex formation is not clear.

**Results:**

We first revisit the basic PDC and show that partial asymptotic phosphorylation of substrate limits ultrasensitivity. Also, substrate levels are changed one can obtain non-monotonic bell-shaped dose-response curves over a narrow range of parameters. Then we extend the PDC to include kinase-phosphatase complex formation. We report the possibility of robust bell-shaped dose-response for a specific class of the model with complex formation. Also, we show that complex formation can produce ultrasensitivity outside the Goldbeter-Koshland zero-order ultrasensitivity regime through a mechanism similar to competitive inhibition between an enzyme and its inhibitor.

**Conclusions:**

We conclude that the novel PDC module studied here exhibits new dose-response behaviour. In particular, we show that the bell-shaped response could result in transient phosphorylation of substrate. We discuss the relevance of this result in the context of experimental observations on PI regulation in endosomal trafficking.

## Background

Biochemical networks have a modular structure [1]. The functional modules have different dynamical and input-output properties [2,3]. For example positive feedback loops can produce bistability while negative feedback loops filter noise. An important biochemical module in cellular signalling is a phosphorylation-dephosphorylation cycle (PDC). Phosphorylation is a common post-translational covalent modification of proteins and lipids, that is mediated by kinases and needs ATP to proceed. However, dephosphorylation is mediated by phosphatases and does not need ATP to proceed. Phosphorylation can affect binding properties, localization and activity of proteins and receptors [4].

Systems with phosphorylation-dephosphorylation cycles can exhibit a variety of input-output or dose-response behaviors [5]. The level of phosphorylated substrate at steady-state is controlled by the kinase-phosphatase balance (KPB), i.e., the ratio of total active kinase to active phosphatase concentration. If the enzymes are far from saturation, the phosphorylated level of substrate is a graded function of the KPB. However, if the enzymes are saturated and are operate in zero-order regime, an ultrasensitive switch-like response is achieved where a small change in the KPB can produce a large change in the level of phosphorylated substrate [6]. Small modifications to the structure of these biochemical modules, such as the introduction of cooperativity or product inhibition can significantly affect their dynamical properties [7,8]. Cascades of phosphorylation-dephosphorylation cycles such as MAP kinase cascades also produce ultrasensitivity and amplification [5]. Multisite protein phosphorylation with distributive mechanism can produce robust ultrasensitivity [9]- [11]. Systems with multiple phosphorylation sites can exhibit additional dynamical properties including multistability [12,13]. Multisite phosphorylation can also result in robust ultrasensitivity outside zero-order regime through local saturation [14,15]. In addition, the order and the distributivity of phosphorylation affects the response properties of the system [4].

**Figure 1:**
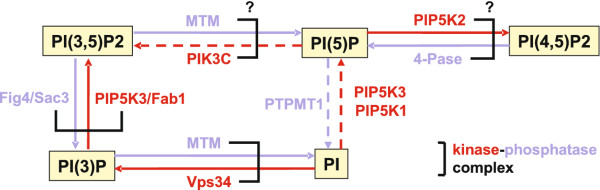
**Kinase-phosphatase complexes.** Identified or hypothesized kinase-phosphatase complexes in the PI regulation system. Full line represents *in vivo* and dashed line *in vitro* evidence. The data is based on the reference [22].

During endosome trafficking tagging molecules that specify the identity and the fate of different vesicles need to be tightly regulated in time. Two classes of molecules that have a fundamental role as molecular tags in membrane traffic are small GTPases and phosphopinositides (PIs) [16]. PIs are a type of cellular phospholipids that can undergo phosphorylation-dephosphorylation cycles. Although PIs are present at relatively low abundance, they have important roles in cellular regulation and membrane trafficking [17,18]. In particular, PIs can get phosphorylated at positions 3, 4 and 5 of their inositol headgroup, through organelle specific kinases and phosphatases [17,18]. Interestingly, several antagonistic kinase and phosphatase pairs regulating phosphorylation in PIs form complexes (Figure 1). Since these kinsase-phosphatase complex pairs have opposing effects, the formation of this kind of complexes between them is puzzling and their functional significance is not clear. For example, the Vps34 kinase generates PI(3)P on endosomes by phosphorylating the third position of the PI inositol headgroup and is regulated by Vps15 protein kinase [19]. Cao *et al*. [20,21] demonstrated that Vps34-Vps15 subcomplex could also interact *in vivo* and *in vitro* with the direct antagonist MTM1 and MTMR2 phosphatases, where Vps15 mediates the interaction between the Vps34 and the myotubularin isoforms.

Another example of an antagonistic kinase-phosphatase pair in yeast and mammals is the Fab1 PI(5)P 5-kinase (also called PIP5K3 or ’PIKfyve’) and the Fig4 PI(3,5)P_2_5-phosphatase (also called Sac3) [23]- [25]. Moreover, the existence of two new kinase-phosphatase complexes which play a role in the regulation of PI(5)P has also been hypothesized [22]. In particular, it has been proposed that a PIK3C (class-I PI 3-kinase) and myotubularin complex regulates the interconversion between PI(3,5)P_2 _and PI(5)P [22]. Less is known about the PIP5K2 4-kinases and PI(4,5)P_2 _4-phosphatases which regulate the interconversion between PI(4,5)P_2 _and PI(5)P, however, the existence of a corresponding kinase-phosphatase complex has also been speculated [22]. The identified and hypothesized kinase-phosphatase pairs are summarized in Figure 1. In summary, there is a growing evidence that a number of kinases and phosphatases can form a complex, but the exact role of such a kinase-phosphatase duo remains to be investigated [26]. Moreover, there is some evidence that this kind of complex formation is evolutionarily conserved which suggests functional significance [22]. Appropriate mathematical modelling may shed light on the functional roles of these complexes.

**Figure 2:**
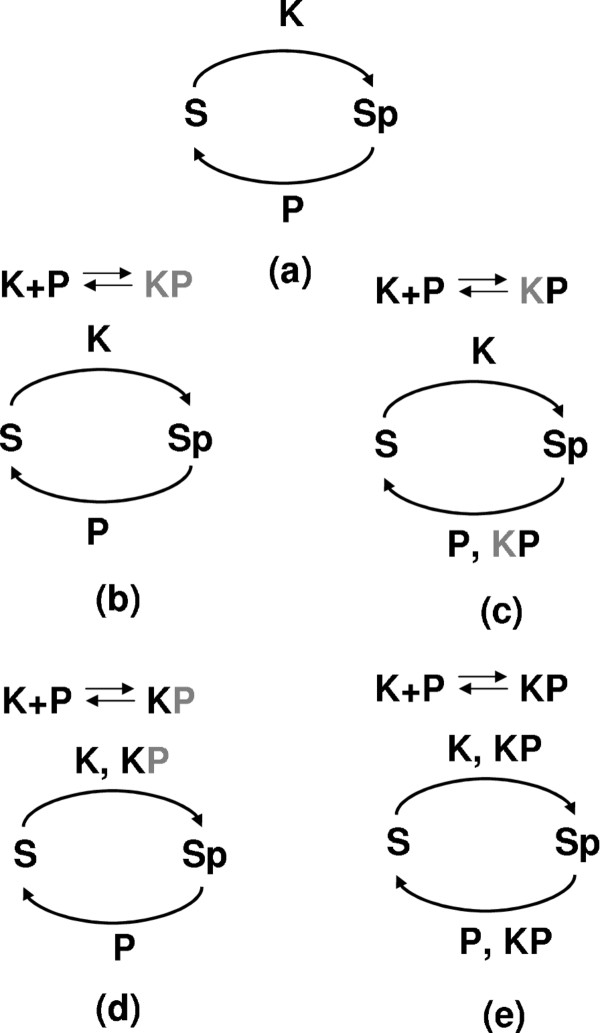
**Reactions diagrams.** (**a**) Reaction diagram for the basic model of phosphorylation-dephosphorylation cycle. (b-e) Reaction diagrams for the extended model with kinase-phosphatase complex formation. The complex can have kinase and/or phosphatase activity. The grey letters in the enzymatic complex name correspond to the inactive enzymes. There are four variants of the extended model with enzymatic complex having both inactive *K* and *P* (**b**), active *P* and inactive *K* (**c**), active *K* and inactive *P* (**d**) and both active *K* and *P* (**e**).

In this paper, we use mathematical modelling to investigate the dose-response in an extended phosphorylation-dephosphorylation cycle that includes complex formation between the kinase and phosphatase. In particular, we ask under what conditions the PDCs can exhibit a non-monotonic dose-response. To this end, we first revisit the basic PDC (Figure 2a) and show that depending on the parameters of the system it is possible to only achieve partial phosphorylation of the substrate even for large KPB. We then use this results to refine estimates of zero-order ultrasensitivity. We further show that at a fix kinase-phosphatase balance, if we change the substrate levels, it is possible to obtain non-monotonic bell-shaped dose-response. However, this behavior is only obtained over a narrow range of parameters.

To investigate the properties of PDC with complex formation, we need to assume enzymatic activity of the kinase-phosphatase complex. Thus, we study four different variants of the extended PDC module, where the complex exhibits kinase and/or phosphatase activity (Figure 2b-e). We observe that the module can produce an ultrasensitive response outside the zero-order regime. In addition, we observe robust bell-shaped non-monotonic dose-responses in a variant of the module where the complex has phosphatase activity. Finally, we discuss our results in the context of PI regulation and endosome trafficking.

## Results and discussion

### Basic model of phosphorylation-dephosphorylation cycle

We shall consider a system in which a substrate can exist in the unphosphorylated form *S* and phosphorylated form *S*_*p*_. The substrate could represent a protein or a PI. A kinase *K* and a phosphatase *P* mediates phosphorylation and dephosphorylation (Figure 2a). *S* and *S*_*p *_are controlled by the following enzymatic reactions:

(1) $$S_{p}+P\overset{\lambda_{1}}{\underset{\lambda_{-1}}{⇌}}S_{p}P\overset{k_{1}}{\to }S+P$$

(2) $$S+K\overset{\lambda_{2}}{\underset{\lambda_{-2}}{⇌}}\text{SK}\overset{k_{2}}{\to }S_{p}+K,$$

where, the rate constants are denoted *λ*_1_,*λ*_2 _for binding an enzyme and substrate, *λ*_−1_,*λ*_−2 _for dissociation of the complex and *k*_1_,*k*_2 _for the catalytic reaction. The association rates (*λ*_*i*_) have units *μ*M^−1^s^−1^, whereas dissociation (*λ*_−*i*_) and catalytic (*k*_*i*_) rates have units s^−1^. The kinetic equations governing the time evolution are

(3) $$\frac{d}{\text{dt}}\left[S_{p}\right]=\lambda_{-1}\left[S_{p}P\right]+k_{2}[\text{SK}]-\lambda_{1}\left[S_{p}\right][P]$$

(4) $$\frac{d}{\text{dt}}\left[S_{p}P\right]=-(\lambda_{-1}+k_{1})\left[S_{p}P\right]+\lambda_{1}\left[S_{p}\right][P]$$

(5) $$\frac{d}{\text{dt}}[S]=\lambda_{-2}[\text{SK}]+k_{1}\left[S_{p}P\right]-\lambda_{2}[S][K]$$

(6) $$\frac{d}{\text{dt}}[\text{SK}]=-(\lambda_{-2}+k_{2})[\text{SK}]+\lambda_{2}[S][K].$$

This system is complemented by conservation equations

(7) $$K_{t}=[K]+[\text{SK}],\;P_{t}=[P]+\left[S_{p}P\right],$$

(8) $$S_{t}=[S]+\left[S_{p}\right]+[\text{SK}]+\left[S_{p}P\right],$$

where the total enzyme *K*_*t*_,*P*_*t *_and substrate *S*_*t *_concentrations are in *μ*M and are often assumed to be constants. At equilibrium,

(9) $$M_{1}\left[S_{p}P\right]=\left[S_{p}\right][P],\;M_{2}[\text{SK}]=[S][K],$$

where the Michaelis constants $M_{i}=\frac{\lambda_{-i}+k_{i}}{\lambda_{i}}$(*i *= 1,2) are in *μ*M.

The system (3)-(8) has been extensively studied over the past 30 years since the key study of zero-order ultra-sensitivity by Goldbeter and Koshland [6]. Most studies look at the steady-state dose-response of the average phosphorylated substrate versus the kinase-phosphatase balance (*K*_*t*_/*P*_*t*_). As a measure of phosphorylated substrate one can either look at the fraction of total phosphorylated substrate (*R *= ([*S*_*p*_] + [*S*_*p*_*P*])/*S*_*t*_) or at the fraction of free phosphorylated substrate (*R*^*′ *^= [*S*_*p*_]/*S*_*t*_). The relevant functional form could be either free or total phosphorylated substrate depending on the specificity of substrate binding sites for phosphatases and recognized targets. In this study, we follow other works that use the fraction of total phosphorylated substrate (*R*) to study the dose response in the PDCs [7,27]. An important approximation used to analyse these systems is the quasi steady-state assumption (QSSA) [28] which assumes that the enzyme-substrate complex concentration remains approximately constant over time (apart from a fast initial transient). Under QSSA, total (*R*) and free (*R*^*′*^) follow the same trends.

Most studies assume that the kinase-phosphatase balance is changed by varying the level of kinase (*K*_*t*_), while keeping the phosphatase levels (*P*_*t*_) constant. This is probably the most relevant regulation that happens in cellular systems. For example, in a MAPK cascade the activity of upstream kinase is increased while effectively the level of phosphatase remains constant. However, this way of varying KPB is asymmetric with respect to *R*. This is why our analysis shows that for low KPB the fraction *R* is always close to zero (no phosphorylation of substrate), however for large KPB the fraction *R* can take any value smaller than one (partial asymptotic phosphorylation of substrate). There are two other ways that KPB can be changed. Another asymmetric method is to keep kinase levels constant and change the level of phosphatase. An example of this kind of regulation has been recently reported in the regulation of yeast mating pathway [14]. A symmetric way of changing KPB is to keep the total level of kinase and phosphatase constant and change the ratio only. This is probably less relevant to cellular systems. However, this way of changing KPB will always result in full asymptotic phosphorylation of substrate at large KPB. Finally, it should be noted that while over some signalling time scales KPB might increase monotonically, over longer time scales there are typically negative feedbacks in place that would decrease KPB, e.g., by increasing the levels of phosphatases [29,30]. In this study, we focus on short-term monotonic changes of KPB ignoring the slow negative-feedback processes.

#### Partial asymptotic phosphorylation of substrate and Hill numbers in the basic model

**Figure 3:**
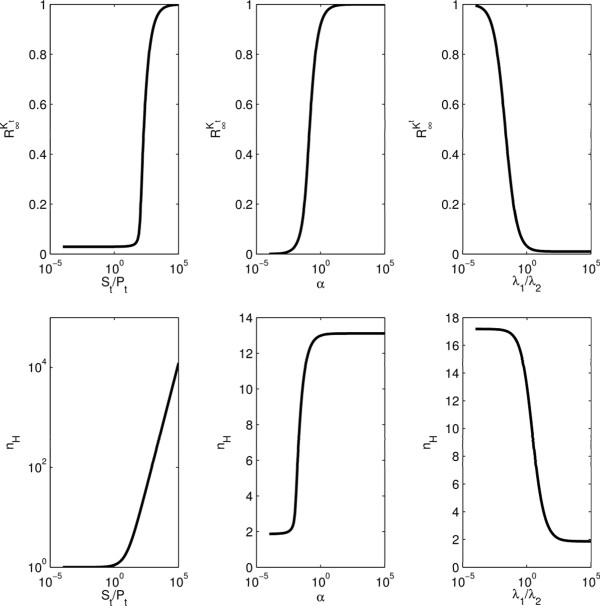
$\left(R_{\infty }^{K_{t}}\right)$**and *n***_***H ***_**for the basic model.** (**a**) $\left(R_{\infty }^{K_{t}}\right)$ is computed analytically as *S*_*t*_/*P*_*t*_, *α*and *λ*_1_/*λ*_2 _is varied. Baseline parameter values used for the basic model, are: *λ*_−1 _= 1 s^−1^,*λ*_1 _= 1 M^−1^s^−1^,*k*_1 _= 1 s^−1^,*λ*_−2 _= 1 s^−1^,*λ*_2 _= 1*μ*M^−1^s^−1^,*k*_2 _= 0*.*01 s^−1^,*P*_*t *_= 1*μ*M,*S*_*t *_= 10*μ*M*.*(**b**) *n*_*H *_is computed analytically as *S*_*t*_/*P*_*t*_, *α*and *λ*_1_/*λ*_2_is varied. Baseline parameter values used for the basic model are: *λ*_−1 _= 1 s^−1^,*λ*_1 _= 1*μ*M^−1^s^−1^,*k*_1 _= 1 s^−1^,*λ*_−2 _= 1 s^−1^,*λ*_2 _= 1*μ*M^−1^s^−1^,*k*_2 _= 1 s^−1^,*P*_*t *_= 1*μ*M,*S*_*t *_= 100*μ*M.

Under QSSA, the asymptotic limit of *R* for large kinase-phosphatase balance (e.g., as *K*_*t *_→ *∞*), denoted by $\left(R_{\infty }^{K_{t}}\right)$ is always one, meaning that the substrate gets fully phosphorylated. However, this is not the case in general when QSSA is not valid, as shown using the exact steady-state solution of (3)-(8) (see Methods). In fact, the asymptotic phosphorylation can be very small for some parameter values as seen using the analytically calculated $\left(R_{\infty }^{K_{t}}\right)$ dependence on the relevant parameter ratios *S*_*t*_/*P*_*t*_,*α*,*λ*_1_/*λ*_2 _in Figure 3(a). Intuitively, the substrate is only partially asymptotically phosphorylated when the phosphatase can sequester a significant fraction of substrate by either saturating the substrate (small $\frac{S_{t}}{P_{t}}$), having higher catalytic rate (small $\alpha =\frac{k_{2}}{k_{1}}$), or having a higher affinity for substrate than kinase (large $\frac{\lambda_{1}}{\lambda_{2}}$) (Figure 3(a)) [31]. Similar results have been previously obtained for other protein modification cycles [32]. The observed limits in Figure 3(a) are $\left(R_{\infty }^{K_{t}}\to 1\right)$ for *S*_*t*_/*P*_*t *_→ *∞* and $\left(R_{\infty }^{K_{t}}\to \alpha /(\alpha +1)\right)$ for *λ*_1_/*λ*_2_→*∞*; these results are obtained in the Methods section.

Goldbeter and Koshland have introduced analytical formulas for the response coefficient (which is defined as the ratio of the kinase concentration required to give 90% response relative to the concentration required to give 10% response; assuming constant phosphatase concentration), both using QSSA and the exact steady-state solution. These results, however, do not take into account the limit of *R* which in general can be smaller than one and based on which the 10% and 90% value should be calculated. Here, we have used results for *R* obtained analytically to introduce a new response coefficient formula and hence, to find the Hill-number (see methods). To illustrate our analysis, we have looked at the dependence of the analytically calculated *n*_*H*_with respect to relevant parameter ratios *S*_*t*_/*P*_*t*_,*α*,*λ*_1_/*λ*_2 _(Figure 3(b)). Comparison with numerically obtained values of $\left(R_{\infty }^{K_{t}}\right)$ and *n*_*H*_(obtained by fitting a Hill function; not shown) confirms our analytical results. As expected, *n*_*H*_is very large at high substrate to phosphatase concentration ratio (zero-order regime; Figure 3(b)). Interestingly, large changes in *n*_*H*_are also obtained when *α *and *λ*_1_/*λ*_2 _are varied. Comparing results in Figure 3(a) and Figure 3(b) suggests that high Hill numbers are only achieved when $\left(R_{\infty }^{K_{t}}\right)$ is close to zero. However, modest ultrasensitivity is present even at very low asymptotic phosphorylation levels of substrate. These results are also consistent with another work which suggests that substrate sequestration can significantly reduce ultrasensitivity [31].

#### Bell-shaped dose-response in the basic model

Most biochemical signalling that involves phosphorylation is mediated by changes of KPB by either regulating the level of active kinase or active phosphatase as explained above. However, one can imagine cases where the substrate level can change, while the KPB is relatively constant. Therefore, it is interesting to ask what kind of dose-response one can expect when the substrate levels are changed.

**Figure 4:**
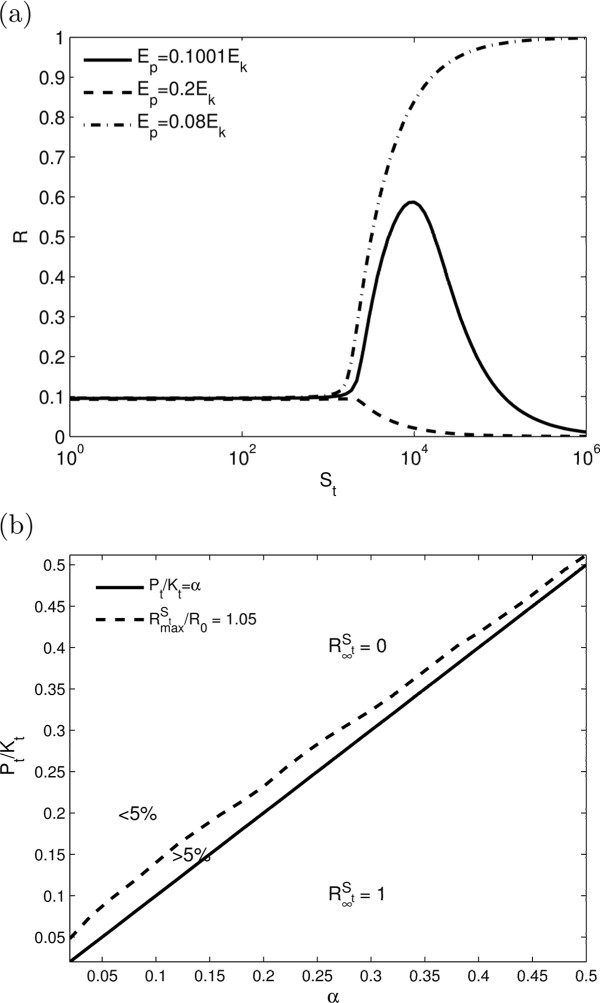
**Bell-shaped dose-response for the basic model.** (**a**) Bell-shaped dose response for the basic model when *S*_*t *_is varied. Parameters used are: *λ*_−1 _= *λ*_1 _= *λ*_2 _= *λ*_−2 _= 0*.*1s^−1^,*k*_1 _= 1 s^−1^,*k*_2 _= 0*.*1s^−1^,*K*_*t *_= 2000*μ*M,*E*_*p *_= 0*.*1001*E*_*k*_(−),*E*_*p *_= 0*.*2*E*_*k*_(−−),*E*_*p *_= 0*.*08*E*_*k*_(−*.*) (**b**) 2D parameter plot when *α*and *P*_*t*_/*K*_*t *_are varied. Parameters used are *λ*_−1 _= *λ*_1 _= *λ*_2 _= *λ*_−2 _= 0*.*1 s^−1^,*α *= 0*.*1,*K*_*t *_= 2000*μ*M*.*$\left(R_{\max}^{S_{t}}\right)$ denotes the maximum of *R*with respect to *S*_*t *_and $\left(R_{0}=\underset{S_{t}\to 0_{+}}{\text{lim}}R\right)$. Within a narrow region above the line *P*_*t*_/*K*_*t *_= *α *the ratio $R_{\max}^{S_{t}}/R_{0}>1.05$, i.e., there is at least a 5% increase in $R_{\max}^{S_{t}}/R_{0}$ compared to *R*_0_.

To address this question we introduce asymptotic phosphorylation of substrate for large substrate levels (as *K*_*t *_→ *∞*) denoted by $\left(R_{\infty }^{S_{t}}\right)$. As shown in the Methods, when the maximum speed of kinase activity is greater than the maximum speed of phosphatase activity ( $\frac{P_{t}}{\alpha }<K_{t}$), the asymptotic phosphorylation is $\left(R_{\infty }^{S_{t}}=1\right)$, since kinase activity wins at large *S*_*t*_. For this case we observe a monotonic increasing dose-response with mild ultrasensitivity with Hill coefficients about 2 (Figure 4(a)). More interesting behaviour is achieved for the case when the maximum speed of kinase activity is less than the maximum speed of phosphatase activity ( $\frac{P_{t}}{\alpha }>K_{t}$) and the asymptotic phosphorylation is $\left(R_{\infty }^{S_{t}}=0\right)$. In this case, there are specific parameters, where a bell-shaped non-monotonic dose-response can be observed (Figure 4(a)). As *S*_*t *_is increased from zero, *R* is initially a constant that is set by the KPB. For large *S*_*t*_, it eventually approaches its asymptote of zero. For most parameters, the transition from constant *R* to zero is monotonic (Figure 4(a)). However, as shown in Figure 4(b), over a narrow region of parameters above the line *P*_*t*_/*K*_*t *_= *α*, it can exhibit a significant non-monotonic bell-shaped response. The bell-shaped response is produced as the transition from $\left(R_{\infty }^{S_{t}}=0\right)$ to $\left(R_{\infty }^{S_{t}}=1\right)$ is approached at *P*_*t*_/*K*_*t *_= *α* (See Eq. 26 in the Methods). Since, this behaviour is only achieved with fine tuning of the parameters and therefore is not expected to be of biological relevance.

### Model with kinase-phosphatase complex formation

In this section we consider an extension to the basic model of phosphorylation-dephosphorylation cycle (Figure 2). We assume that the phosphatase and kinase can form a single protein complex *PK* given through a reversible reaction (10):

(10) $$P+K\overset{\kappa_{1}}{\underset{\kappa_{-1}}{⇌}}\text{PK}$$

Depending on which enzyme is ’on’ or ’off’ within the kinase-phosphatase complex, the substrates *S*/*S*_*p*_could be further phosphorylated/dephosphorylated by *PK* as shown in (11) and (12):

(11) $$S_{p}+\text{PK}\overset{\lambda_{3}}{\underset{\lambda_{-3}}{⇌}}S_{p}\text{PK}\overset{k_{3}}{\to }S+\text{PK}$$

(12) $$S+\text{PK}\overset{\lambda_{4}}{\underset{\lambda_{-4}}{⇌}}S_{p}\text{PK}\overset{k_{4}}{\to }S_{p}+\text{PK}$$

In (10)-(12), the parameters *κ*_1_,*λ*_3_,*λ*_4 _and *κ*_−1_,*λ*_−3_,*λ*_−4 _correspond to the association/dissociation rates, respectively, and *k*_3_,*k*_4 _represent the catalytic reactions. Here we define a parameter *ω *= *κ*_−1_/*κ*_1_, similar to the concept of a ’dissociation constant’, which expresses the affinity of binding between the phosphatase *P* and kinase *K*.

There are four possibilities as shown in Figure 2; both enzymes in the complex could be enzymatically inactive (Figure 2(b)), only phosphatase active (Figure 2(c)), only kinase active (Figure 2(d)) or both kinase and phosphatase active (Figure 2(e)). This system is described by the following kinetic equations

(13) $$\begin{array}{l} \frac{d}{\text{dt}}\left[S_{p}\right]=\lambda_{-1}\left[S_{p}P\right]+k_{2}[\text{SK}]-\lambda_{1}\left[S_{p}\right][P]\\ \;\;\;+\lambda_{-3}\left[S_{p}\text{PK}\right]-\lambda_{3}\left[S_{p}\right][\text{PK}]+k_{4}[\text{SPK}] \end{array}$$

(14) $$\frac{d}{\text{dt}}\left[S_{p}P\right]=-(\lambda_{-1}+k_{1})\left[S_{p}P\right]+\lambda_{1}\left[S_{p}\right][P]$$

(15) $$\begin{array}{l} \frac{d}{\text{dt}}[S]=\lambda_{-2}[\text{SK}]+k_{1}\left[S_{p}P\right]-\lambda_{2}[S][K]+k_{3}\left[S_{p}\text{PK}\right]\\ \;\;+\lambda_{-4}[\text{SPK}]-\lambda_{4}[S][\text{PK}] \end{array}$$

(16) $$\begin{array}{ll} \frac{d}{\text{dt}}[\text{SK}]=-(\lambda_{-2}+k_{2})[\text{SK}]+\lambda_{2}[S][K] & \; \end{array}$$

(17) $$\begin{array}{l} \frac{d}{\text{dt}}[\text{PK}]=\kappa_{1}[P][K]-\kappa_{-1}[\text{PK}]+(\lambda_{-3}+k_{3})\left[S_{p}\text{PK}\right]\\ \;\;\;-\lambda_{3}\left[S_{p}\right][\text{PK}]+(\lambda_{-4}+k_{4})[\text{SPK}]-\\ \;\;\;-\lambda_{4}[S][\text{PK}] \end{array}$$

(18) $$\begin{array}{ll} \frac{d}{\text{dt}}\left[S_{p}\text{PK}\right]=\lambda_{3}\left[S_{p}\right][\text{PK}]-(\lambda_{-3}+k_{3})\left[S_{p}\text{PK}\right] & \; \end{array}$$

(19) $$\begin{array}{ll} \frac{d}{\text{dt}}[\text{SPK}]=\lambda_{4}[S][\text{PK}]-(\lambda_{-4}+k_{4})[\text{SPK}] & \; \end{array}$$

coupled with conservation equations

(20) $$\begin{array}{cc} \left[P\right]+\left[S_{p}P\right]+\left[S_{p}\text{PK}\right]+[\text{PK}] & =P_{t},\\ \left[K]+[\text{SK}]+[\text{SPK}]+[\text{PK}\right] & =K_{t}, \end{array}$$

(21) $$\begin{array}{ll} \;\left[S_{p}\right]+[S]+\left[S_{p}P\right]+[\text{SK}]+\left[S_{p}\text{PK}\right]+[\text{SPK}]=S_{t}. & \; \end{array}$$

We note that the the extended model (13)-(21) is too complicated to find the corresponding steady-states analytically. In the following we have used numerical integration of the kinetic equations above and variants of the QSSA to study the dose-response in this system. The ration of phosphorylated substrate in the extended model is now defined as *R*=([*S*_*p*_] + [*S*_*p*_*P*] + [*S*_*p*_*PK*])/*S*_*t*_. Our goal is to determine the dose-response for *R* as a function of KPB in the extended model. We have mainly focused on the kinase active case and the phosphatase active case which are the two extreme scenarios (Figure 2(c)-(d)).

#### Ultrasensitivity

**Figure 5:**
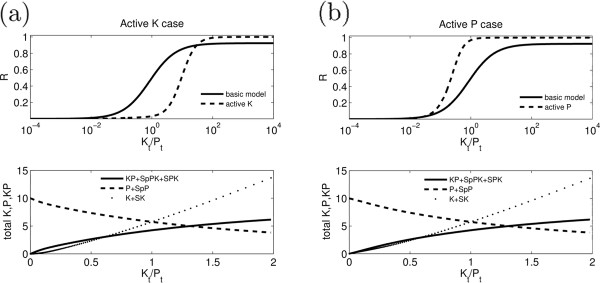
**Ultrasensitivity.** (**a**) *R*, total *K*, *P* and *KP* are numerically calculated as KPB is changed in the extended model with only kinase active (Figure 2d). Parameters used are: *λ*_−1 _= 10 s^−1^,*λ*_1 _= 1*μ*M^−1^s^−1^,*k*_1 _= 100 s^−1^,*λ*_−2 _= 10 s^−1^,*λ*_2 _= 1*μ*M^−1^s^−1^,*k*_2 _= 100 s^−1^,*λ*_−4 _= 10 s^−1^,*λ*_4 _= 100*μ*M^−1^s^−1^,*k*_4 _= 0*.*01 s^−1^,*κ*_−1 _= 10 s^−1^,*κ*_1 _= 1*μ*M^−1^s^−1^,*P*_*t *_= 10*μ*M,*S*_*t *_= 1*μ*M*.*Hill coefficient for basic model and active *K* is *n*_*H *_= 1 and *n*_*H *_= 1*.*8, respectively. (**b**) *R*, total *K*, *P* and *KP* are numerically calculated as KPB is changed in the extended model with only phosphatase active (Figure 2c). Parameters used are: *λ*_−1 _= 10 s^−1^,*λ*_1 _= 1*μ*M^−1^s^−1^,*k*_1 _= 100 s^−1^,*λ*_−2 _= 10 s^−1^,*λ*_2 _= 1*μ*M^−1^s^−1^,*k*_2 _= 100 s^−1^,*λ*_−3 _= 10 s^−1^,*λ*_3 _= 100*μ*M^−1^s^−1^,*k*_3 _= 0*.*01 s^−1^,*κ*_−1 _= 10 s^−1^,*κ*_1 _= 1*μ*M^−1^s^−1^,*P*_*t *_= 10*μ*M,*S*_*t *_= 1*μ*M. Hill coefficient for basic model and active *K* is *n*_*H *_= 1 and *n*_*H *_= 1*.*95, respectively. In both case the bottom panels show that the level of enzymes remains above the substrate, so we are far from the zero-order regime.

We observe ultrasensitivity for both the kinase active-phosphatase inactive and the phosphatase active-kinase inactive cases outside the zero-order regime, where *S*_*t *_<*P*_*t *_(Figure 5). For the phosphatase active case, the mechanism is similar to the previously described reversible competitive inhibition, where the substrate and the inhibitor cannot bind the enzyme at the same time [5]. In this case, when the level of *K* is increased, initially it binds to *P* and forms *KP* that acts as a phosphatase and *R* remains close to zero. However, at some level of *K*, most of the free *P* will be in complex with *K*. Above this level a small increase in *K* changes the phosphorylation level of substrate (*R*) nonlinearly, producing ultrasensitivity.

The mechanism of ultrasensitivity for the kinase active case is novel. In this case, when the level of *K* is increased, it binds to *P* and forms *KP* that acts as a kinase. Therefore, increasing levels of *K* has the compound effect of increasing the kinase activity and at the same time reducing the phosphatase activity, resulting in a nonlinear increase in *R* and an ultrasensitive response. As shown in the Figure 5, both ultrasensitivity mechanisms are independent of the enzyme saturation and can produce sharp responses in the parameter regime where the basic model cannot.

#### Bell-shaped dose-response curves

The active phosphatase case, when the kinase is ’off’ and the phosphatase is ’on’ in the protein complex is interesting since it can exhibit a non-monotonic response. Suppose that the affinity between *P* and *K* is low and the *PK* complex is a stronger phosphatase than *P* alone (with a lower $\left(R_{\infty }^{K_{t}}\right)$). In this case, when the level of *K* is increased initially, the *KP* production is small. However, the kinase activity soon takes over the phosphatase activity and *R* is increased close to $\left(R_{\infty }^{K_{t}}\right)$. As *K* is increased, the *KP* production becomes more dominant, and, since *KP* is a stronger phosphatase, the $\left(R_{\infty }^{K_{t}}\right)$ is dropped to a lower level, hence, producing a bell-shaped response. As *K*_*t *_→ *∞*, the limit of the response curve has been derived earlier for the basic model. This measure can give an estimate of the peak level of *R*. However, this $\left(R_{\infty }^{K_{t}}\right)$-formula could be used for the active *P* case as well. Indeed, at high *K*_*t *_concentrations the free phosphatase is close to zero and we effectively reduce to the basic model with only *K* and *PK* around. Hence, replacing the parameters *λ*_1_,*λ*_−1_,*k*_1 _by *λ*_3_,*λ*_−3_,*k*_3 _in (27) gives the desired result for the asymptotic level of *R*.

**Figure 6:**
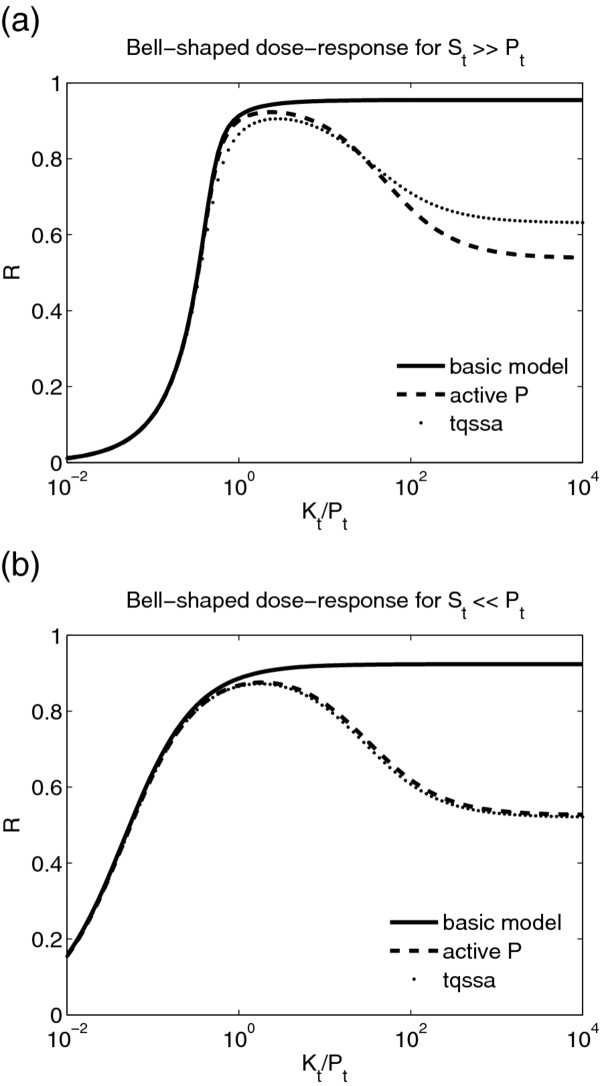
**Bell-shaped dose-response in the active phosphatase case (Figure**2**c).** (**a**) Bell-shaped response in the zero-order regime when *S*_*t *_≫ *P*_*t*_. Parameters used are: *λ*_−1 _= 10 s^−1^,*λ*_1 _= 1*μ*M^−1^s^−1^, *k*_1 _= 1 s^−1^,*λ*_−2 _= 10 s^−1^,*λ*_2 _= 20*μ*M^−1^s^−1^,*k*_2 _=1 s^−1^,*λ*_−3 _= 10 s^−1^,*λ*_3 _= 1*μ*M^−1^s^−1^,*k*_3 _= 100*c*s^−1^,*κ*_−1 _= 50 s^−1^,*κ*_1 _= 1*μ*M^−1^s^−1^,*P*_*t *_= 1*μ*M,*S*_*t *_= 10*μ*M. The Hill coefficient for the basic model is *n*_*H *_= 2*.*15. (**b**) Bell-shaped response outside the zero-order regime when *S*_*t *_≪ *P*_*t*_: *λ*_−1 _=10 s^−1^,*λ*_1 _= 1*μ*M^−1^s^−1^,*k*_1 _= 1 s^−1^,*λ*_− 2_= 10 s^−1^,*λ*_2 _= 20*μ*M^−1^s^−1^,*k*_2 _= 1 s^−1^,*λ*_−3 _= 10 s^−1^,*λ*_3 _= 1*μ*M^−1^s^−1^,*k*_3 _= 100 s^−1^,*κ*_−1 _= 50 s^−1^,*κ*_1 _= 1*μ*M^−1^s^−1^,*P*_*t *_= 1*μ*M,*S*_*t *_= 0*.*1*μ*M. The Hill coefficient for the basic model is *n*_*H *_= 1. In (a)-(b), the full line corresponds to the basic model, the dashed line is numerical results for the extended model and the dotted line shows the tQSSA approximation.

Bell-shaped responses could be obtained both at high enzyme saturation (*S*_*t *_≫ *P*_*t*_), and at low enzyme saturation (*S*_*t *_≪ *P*_*t*_). Figures 6(a)-(b) compare the response curve for the active *P* with the response curve for the corresponding basic model for both scenarios. The only difference is that for the low enzyme saturation we have an ultrasensitive rise to the peak of the bell-shaped response, whereas for the high enzyme saturation there is a linear rise to the peak.

The steady-state solutions of the extended model can only be defined implicitly. Hence, we can use the total quasi-steady-state-approximation (tQSSA), which is more generally valid than the QSSA [33,34]. The tQSSA method has been successfully applied to a model with a pair of enzymes and substrates (analogous to the basic PDC) [27] and to coupled PDCs [35]. The details of the tQSSA method are explained in the Methods section. The results of tQSSA calculation is consistent with the numerical results in Figures 6(a)-(b). The fact that tQSSA but not QSSA can explain the bell-shaped response suggests that the dynamic of enzyme-substrate complex contributes to the presence of the non-monotonic response.

**Figure 7:**
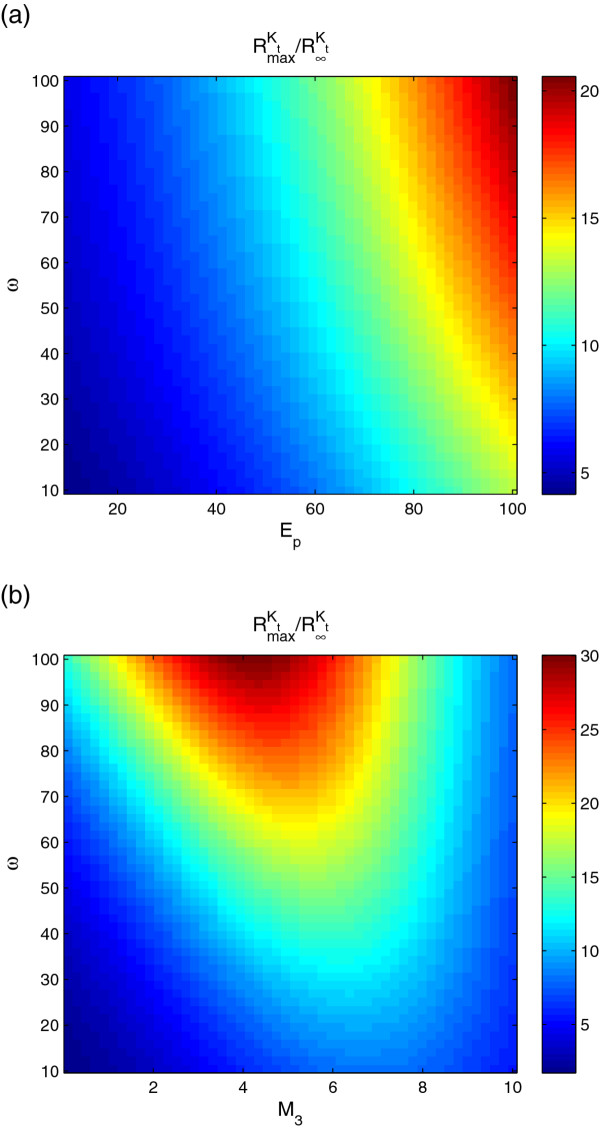
**Robustness of the bell-shaped response.** To illustrate the robustness of the bell-shaped response, the ratio of $\left(R_{\max}^{K_{t}}\right)$, the maximum of *R* with respect to *K*_*t*_, to $\left(R_{\infty }^{K_{t}}\right)$ is calculated as multiple parameters are varied. The red regions correspond to parameter values with the largest bell-shaped response. (**a**) *ω *and *P*_*t *_are varied (*κ*_−1_as a variable in *ω*). (**b**) *ω *and *M*_3 _are varied (*κ*_−1 _as a variable in *ω*and *λ*_3 _as a variable in *M*_3_). Parameter values used in the extended model with only phosphatase active in (a) and (b): *λ*_−1 _= 10 s^−1^,*λ*_1 _= 1*μ*M^−1^s^−1^,*k*_1 _= 1 s^−1^,*λ*_−2 _= 10 s^−1^,*λ*_2 _= 20*μ*M^−1^s^−1^,*k*_2 _= 1 s^−1^,*λ*_−3 _= 10 s^−1^,*λ*_3 _= 1*μ*M^−1^s^−1^,*k*_3 _= 100 s^−1^,*κ*_−1 _= 10 s^−1^,*κ*_1 _= 1*μ*M^−1^s^−1^,*P*_*t *_= 1*μ*M,*S*_*t *_= 10*μ*M.

**Figure 8:**
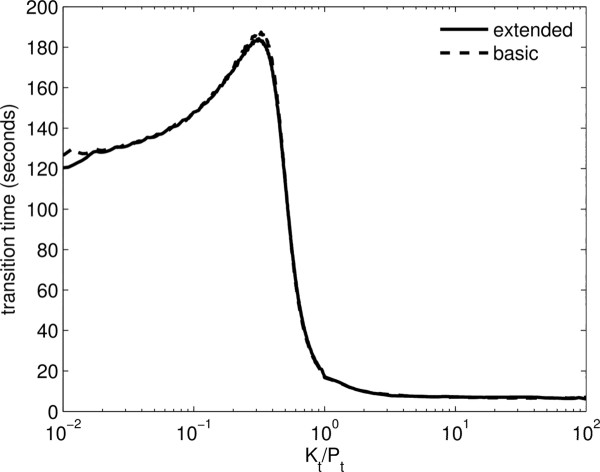
**Transient times to approach steady-state for the basic and extended model.** Parameter values for the basic model: *λ*_−1 _= 10 s^−1^,*λ*_1 _= 1*μ*M^−1^s^−1^,*k*_1 _= 1 s^−1^,*λ*_−2 _= 10 s^−1^,*λ*_2 _= 20*μ*M^−1^s^−1^,*k*_2 _= 1 s^−1^,*P*_*t *_= 1*μ*M,*S*_*t *_= 10*μ*M*.*Parameter values for the extended model: *λ*_−1 _= 10 s^−1^,*λ*_1 _= 1*μ*M^−1^s^−1^,*k*_1 _= 1 s^−1^,*λ*_−2 _= 10 s^−1^,*λ*_2 _= 20*μ*M^−1^s^−1^,*k*_2 _= 1 s^−1^,*κ*_−1 _= 50 s^−1^,*κ*_1 _= 1*μ*M^−1^s^−1^,*λ*_−3 _= 10 s^−1^,*λ*_3 _= 1*μ*M^−1^s^−1^,*k*_3 _= 100 s^−1^,*P*_*t *_= 1*μ*M,*S*_*t *_= 10*μ*M*.*The transient times for *R *= ([*S*_*p*_] + [*S*_*p*_*P*] + [*S*_*p*_*PK*])/*S*_*t *_were estimated such that the absolute difference between the positive steady-state and the time solution at the transient time is within a small tolerance and that the transient times do not change too much if the tolerance is varied. Hence, we chose the tolerance to be 10^−4^.

We have investigated the robustness of the bell-shaped response by varying some of the key parameters. We have characterised the bell-shape response by looking at the ratio of the maximum response to asymptotic phosphorylation as a function of dissociation constant of the phosphatase-kinase complex $\omega =\frac{\kappa_{-1}}{\kappa_{1}}$, the Michaelis-Menten constant $M_{3}=\frac{\lambda_{-3}+k_{3}}{\lambda_{3}}$ of the complex and the total phosphatase level *P*_*t*_(Figure 7). It is possible to obtain significant bell-shaped responses over a large range of parameters. The response has the highest relative peak for large values of *ω *and *P*_*t*_(Figure 7(a)), however for each parameter set there is an optimal value of *M*_3_(Figure 7(b)). Also, we have compared the transient time needed to approach steady-state in the extended model with active *P* (Figure 8). We observe that the transient time for the extended model follows closely the transient time for the basic model and it goes through a maximum at the threshold of ultrasensitivity. This is because the enzymatic complex formation is fast compared to phosphorylation and dephosphorylation.

### Possible role for complex formation in phosphoinositides regulation

We propose that complex formation can contribute to the transient regulation of PIs observed experimentally in endosome trafficking [36]. Suppose the kinase is being recruited to the vesicle with a constant rate (slower than the time scale for the transient behaviour in Figure 8) and there is a fixed level of phosphatase on the vesicle. This produces a gradual change in KPB. If there is complex formation and the complex has phosphatase activity, in the parameter regime where we have bell-shaped dose-response as discussed above, the level of phosphorylated PI goes through a maximum as KPB is changed over time. This can produce a fast and accurate transient change in the phosphorylation of PI.

Botelho [37] has speculated on the possible role of enzymatic complex formation in transient regulation of PIs but he has proposed an alternative mechanism to achieve a transient behaviour. This alternative explanation assumes that there is a signaling trigger that activates the phosphatase relative to the kinase in the enzymatic complex leading to the down-regulation of phosphorylated PI on the membrane. Therefore enzymatic activity of the complex switches from kinase to phosphatase upon signalling. The signalling trigger might be the recruitment of other vesicles or GTP hydrolysis by GTPases [37]. The Fab1-Figure 4 and Vps34-MTM complexes in PI regulation could follow this concept (Figure 1).

**Figure 9:**
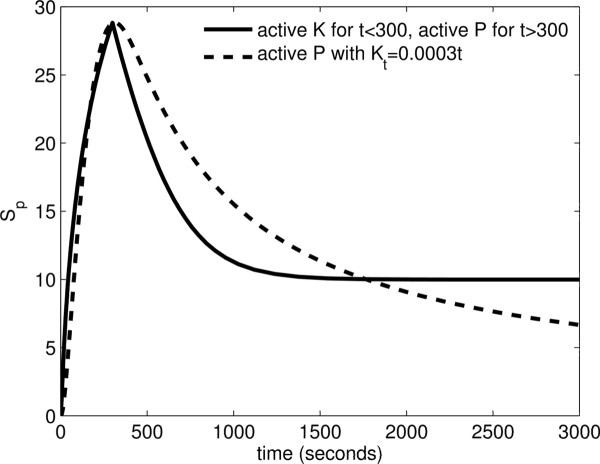
**Transient phosphorylation of phosphoinositides due to enzymatic complex formation.** Comparison of the two scenarios; solid line represent the case when a signalling trigger is simulated. Parameters used are the following: *λ*_−1 _= 10 s^−1^,*λ*_1 _= 1*μ*M^−1^s^−1^,*k*_1 _= 1 s^−1^,*λ*_−2 _= 10 s^−1^,*λ*_2 _= 1*μ*M^−1^s^−1^,*k*_2 _= 1 s^−1^,*κ*_−1_=50s^−1^,*κ*_1 _= 1*μ*M^−1^s^−1^,*K*_*t *_= 0*.*1*μ*M,*P*_*t *_= 0*.*2*μ*M,*S*_*t *_= 300*μ*M*.*The other parameters are *λ*_−4 _= 1 s^−1^,*λ*_4 _= 75*μ*M^−1^s^−1^,*k*_4 _= 100 s^−1^for *t*≤300 s and *λ*_−3 _= 10 s^−1^,*λ*_3 _= 10*μ*M^−1^s^−1^,*k*_3 _= 500 s^−1^, for *t*>300s. Dashed line represent the case when kinases are recruited linearly in time as *K*_*t *_= 0*.*0003*t*. Other parameters used are the following: *λ*_−1 _= 10 s^−1^,*λ*_1 _= 1*μ*M^−1^s^−1^,*k*_1 _= 1 s^−1^,*λ*_−2 _= 10 s^−1^,*λ*_2 _= 1*μ*M^−1^s^−1^,*k*_2 _= 1 s^−1^,*κ*_−1 _= 50 s^−1^,*κ*_1 _= 1*μ*M^−1^s^−1^,*λ*_−3 _= 10 s^−1^,*λ*_3 _= 10*μ*M^−1^s^−1^,*k*_3 _= 500 s^−1^,*P*_*t *_= 0*.*2*μ*M,*S*_*t *_= 300*μ*M.

In Figure 9 we have compared the time evolution of phosphorylated substrate (PI) following the two alternative mechanisms mentioned above. In the first scenario, we assume a hypothetical recruitment of kinases over time to the membrane, fixed levels of phosphatase on the membrane and the active phosphatase activity in the *PK* complex. In the second scenario following Botelho [37], we assume a fixed level of kinase and phosphatase and we assume a switch in the enzymatic activity of the *PK* complex from kinase to phosphatase at *t *= 300 s. Both scenarios can result in fast transient phosphorylated substrate which peaks at 300 seconds, while our proposed mechanism results in faster decay as *K*_*t *_increases over time. While both phosphorylation profiles in Figure 9 are in qualitative agreement with the observed profiles in the process of endocytosis [36], however, the dynamic of available kinase is quite different in the two models and can be used to experimentally test our proposed hypothesis. Note that in our proposed mechanism, the asymptotic phosphorylation level of the substrate does not get to zero, however, by increasing the phosphatase activity of the complex, one can make this asymptotic phosphorylation arbitrarily small. Also, a counter intuitive aspect of our proposed model is that if the kinase levels are down-regulated, then the phosphorylation of the substrate could even increase again. So, one requires no down-regulation of the upstream kinase over the relevant time-scales of the transient PI profiles. Ultimately, the response of the PDC module should be considered in the context of the larger PI regulation network (Figure 1) to be able to obtain a full understanding of the role of enzymatic complex formation in the endosome trafficking.

## Conclusions

In this paper, we have studied the steady-state dose-response in basic PDC motif and a modified PDC motif using analytical and numerical methods. We first showed that there are parameter regimes where even at large KPB the phosphorylated ratio of substrate remains small. This is not expected from the QSSA analysis of the basic PDC, and it is a consequence of efficient phosphatase binding to a substrate. This results in modified estimates of the Hill number in zero order ultrasensitivity. In addition, we observe that partial asymptotic phosphorylation of substrate limits the ultrasensitive behavior. Over a narrow parameter regime, one can obtain a bell-shaped dose-response when the substrate level is changed for a fix KPB level. We then studied a modified PDC motif where the antagonistic kinase and phosphatase can form a complex. Depending on the activity of the kinase and phosphatase within the complex, the modified system has four different cases. We investigated the possible dose-response curves in all scenarios. We observed a novel form of ultrasensitivity arising from complex-formation that can function outside the zero-order regime. Also, we report a non-monotonic bell shaped dose-response for the phosphatase active-kinase inactive case that can function over a wide-range of parameters.

Complex formation between antagonistic enzymes have been reported in the PI regulatory network (Figure 1). Motivated by the observed bell shaped dose-response, we propose the complex formation between the kinase and phosphatase may contribute to the observed transient regulation of PIs during endosome maturation [36]. It has been recently proposed that the complex formation observed in several kinase-phosphatase pairs in the PI regulation may enhance the temporal regulation of the PIs [37]. Here we have shown that if the complex has enhanced phosphatase activity and negligible kinase activity, then the dose-response will be bell-shaped. This mechanism produces a transient increase in the phosphorylation level of the PIs as enzymes are recruited to the endosomes and PKB is changed over time. There is no need for additional regulators or triggering mechanisms, but only monotonic change of the PKB on the endosome by enzymatic recruitment. Experimental measurement of enzymatic activity within the complexes are still missing. Our model predicts that an enhanced phosphatase activity within the complex gives rise to bell-shaped dose-response. Also, we predict that alteration of the total level of enzymes or the affinity of enzymes in the complex will have a significant effect on the height of the bell-shaped response.

Our study illustrates that simple alterations of the regulatory motifs in biochemical networks can have a significant consequence on the response and functionality of these systems. Signalling systems are typically composed of several interlinked regulatory motifs. To ultimately understand the function of the whole system one needs to put the motifs together and investigates the coordinated dynamic behaviour of all the components as they interact with one another. In the context of the PI regulation, as shown in Figure 1, there are several PDC motifs with enzymatic complex formation. As shown here, these motifs can produce ultrasensitive or non-monotonic dose-response depending on their biochemical parameters. The challenge that remains is to quantify these parameters and explain the overall function by investigating the interplay between the dynamic of individual motifs.

## Methods

### Limit of the average fraction of phosphorylated substrate for *K*_*t *_→ *∞* and *S*_*t *_→ *∞*

We derive limiting values for the average fraction of phosphorylated substrate *R *= ([*S*_*p*_] + [*S*_*p*_*P*])/*S*_*t*_at steady-state as *K*_*t *_→ *∞* and *S*_*t *_→ *∞*, respectively. First, we will implicitly define the steady-state [*SK*] as a solution of the cubic equation

(22) $${\left[\text{SK}\right]}^{3}+Q_{1}{\left[\text{SK}\right]}^{2}+Q_{2}[\text{SK}]+Q_{3}=0.$$

The coefficients *Q*_*i*_(*i *= 1,2,3) are of the form *Q*_1 _= −*K*_*t*_+ *v*_1_, *Q*_2 _=*u*_1_*K*_*t*_+ *v*_2_, *Q*_3 _=*u*_2_*K*_*t *_with

$$\begin{array}{l} u_{1}=\frac{P_{t}}{\alpha }+\frac{M_{1}+S_{t}}{\alpha +1},\;u_{2}=-\frac{P_{t}S_{t}}{\alpha (\alpha +1)},\\ v_{1}=-\frac{P_{t}}{\alpha }-\frac{M_{1}+M_{2}+S_{t}}{\alpha +1},\;v_{2}=\frac{P_{t}(K_{t}+S_{t})}{\alpha (\alpha +1)}, \end{array}$$

where $\alpha =\frac{k_{2}}{k_{1}}$. It follows from (3) and (9) that *α*satisfies

(23) $$\alpha [\text{SK}]=\left[S_{p}P\right]$$

and hence,

(24) $$[\text{SK}]<\frac{P_{t}}{\alpha }$$

for *P*_*t *_> 0. The asymptotic expansion of [*SK*] with respect to powers of $\frac{1}{K_{t}}$ in (22) implies that the asymptotic limit of [*SK*], i.e., $\left(SK_{\infty }^{K_{t}}={\text{lim}}_{K_{t}\to \infty }[\text{SK}]\right)$ satisfies the quadratic equation

$${\left(SK_{\infty }^{K_{t}}\right)}^{2}-u_{1}SK_{\infty }^{K_{t}}-u_{2}=0.$$

By condition (24) we obtain that

(25) $$SK_{\infty }^{K_{t}}=\frac{u_{1}-\sqrt{u_{1}^{2}+4u_{2}}}{2}.$$

Note that $u_{1}=\frac{P_{t}}{\alpha }+\frac{M_{1}+S_{t}}{\alpha +1}$ and $u_{2}=-\frac{P_{t}S_{t}}{\alpha (\alpha +1)}$ do not depend on *M*_2 _and hence, $\left(SK_{\infty }^{K_{t}}\right)$ does not change when *λ*_2 _or *λ*_−2 _is varied. Intuitively, this is because for high kinase levels the substrate will be saturated and the $\left(SK_{\infty }^{K_{t}}\right)$ will not be influenced by the dissociation constant of kinase-substrate binding. By (9) and (23) the phosphorylated substrate fraction *R* could be re-written as

(26) $$R=\frac{\alpha }{S_{t}}[\text{SK}]\left(\frac{M_{1}}{P_{t}-\alpha [\text{SK}]}+1\right).$$

For *K*_*t *_→ *∞*, the limiting value of *R* denoted by $\left(R_{\infty }^{K_{t}}\right)$ becomes

(27) $$R_{\infty }^{K_{t}}=\frac{\alpha }{S_{t}}SK_{\infty }^{K_{t}}\left(\frac{M_{1}}{P_{t}-\mathrm{\alpha S}K_{\infty }^{K_{t}}}+1\right),$$

where $\left(SK_{\infty }^{K_{t}}\right)$ is given by (25). It is shown in the Methods section (Corollary 1) that *R *≤ 1 as expected.

For *S*_*t *_→ *∞*, we also include a statement about the limiting value of *R* denoted by $\left(R_{\infty }^{S_{t}}\right)$, which will be useful later on (for proof see Corollary 2 in the Methods section). If $\frac{P_{t}}{\alpha }<K_{t}$, then $[\text{SK}]\to \frac{P_{t}}{\alpha }$ and *R *→ 1 as *S*_*t *_→ *∞*, otherwise, [*SK*] → *K*_*t*_and *R *→ 0 as *S*_*t *_→ *∞*. That is,

$$R_{\infty }^{S_{t}}=\left\{\begin{array}{ll} 1 & \text{if}\;\frac{P_{t}}{\alpha }<K_{t}\\ 0 & \text{if}\;\frac{P_{t}}{\alpha }>K_{t}. \end{array}\right)$$

#### Corollary 1–3

**Corollary 1. ***The ratio R satisfies*

$$R=\frac{\alpha }{S_{t}}[\text{SK}]\left(\frac{M_{1}}{P_{t}-\alpha [\text{SK}]}+1\right)\leq 1.$$

*Proof*. It is sufficient to show that $\left(R_{\infty }^{K_{t}}\leq 1\right)$ since $\frac{\mathrm{dR}}{dK_{t}}=\frac{\mathrm{dR}}{d[\text{SK}]}\frac{d[\text{SK}]}{dK_{t}}>0$ and hence, *R* is monotone increasing with respect to *K*_*t *_and $\left(R_{\infty }^{K_{t}}\right)$ is its maximum. $\left(R_{\infty }^{K_{t}}\leq 1\right)$ is equivalent to the condition

(28) $$\alpha^{2}{\left(SK_{\infty }^{K_{t}}\right)}^{2}-\alpha (M_{1}+P_{t}+S_{t})SK_{\infty }^{K_{t}}+S_{t}P_{t}\geq 0.$$

To show that (28) holds we use that $\left({\left(SK_{\infty }^{K_{t}}\right)}^{2}-u_{1}SK_{\infty }^{K_{t}}-u_{2}=0\right)$ and that $\left(P_{t}>\mathrm{\alpha S}K_{\infty }^{K_{t}}\right)$ (this follows from (24)). Multiply (28) by $\frac{1}{\alpha (\alpha +1)}$ to get

(29) $$\frac{\alpha }{\alpha +1}{\left(SK_{\infty }^{K_{t}}\right)}^{2}-\left(u_{1}-\frac{P_{t}}{\alpha (\alpha +1)}\right)SK_{\infty }^{K_{t}}-u_{2}\geq 0.$$

Note that the left-hand side of (29) is equivalent to

$$SK_{\infty }^{K_{t}}\left(\frac{P_{t}}{\alpha (\alpha +1)}-\frac{SK_{\infty }^{K_{t}}}{\alpha +1}\right)>0$$

and this concludes the proof. □

**Corollary 2. ***If *$\frac{P_{t}}{\alpha }<K_{t}$, then $[\text{SK}]\to \frac{P_{t}}{\alpha }$*and R *→ 1 *as S_t _*→ ∞. *If *$\frac{P_{t}}{\alpha }>K_{t}$, then [*SK*] → *K*_*t*_*and R *→ 0 *as S*_*t *_→ ∞.

*Proof*. Denote $\left(SK_{\infty }^{S_{t}}=\underset{S_{t}\to \infty }{\text{lim}}[\text{SK}]\right)$. It follows from (22) that $\left(SK_{\infty }^{S_{t}}\right)$ satisfies the quadratic equation

$$c_{3}{\left(SK_{\infty }^{S_{t}}\right)}^{2}+(-c_{3}K_{t}+c_{1})SK_{\infty }^{S_{t}}-c_{1}K_{t}=0$$

and then

$$SK_{\infty }^{S_{t}}=\frac{-(-K_{t}-\frac{P_{t}}{\alpha })\pm |-K_{t}+\frac{P_{t}}{\alpha }|}{2}.$$

Thus, $SK_{\infty }^{S_{t}}=\frac{P_{t}}{\alpha }$ if $\frac{P_{t}}{\alpha }<K_{t}$ and $\left(SK_{\infty }^{S_{t}}=K_{t}\right)$ otherwise. Obviously, if $\left(SK_{\infty }^{S_{t}}=K_{t}\right)$, then $\left(R_{\infty }^{S_{t}}=0\right)$. To see that if $SK_{\infty }^{S_{t}}=\frac{P_{t}}{\alpha }$, then $\left(R_{\infty }^{S_{t}}=1\right)$, we show that [*Sp*]→*S*_*t*_as *S*_*t*_→*∞*. Indeed, note that $[S]=\frac{M_{2}[\text{SK}]}{K_{t}-[\text{SK}]}\to \frac{M_{2}P_{t}}{\alpha K_{t}-P_{t}}$ as *S*_*t*_→*∞*. Hence, [*S*_*p*_]=*S*_*t*_−[*SK*]−[*S*_*p*_*P*]−[*S*]→*S*_*t*_as *S*_*t*_→*∞* and so $\left(R_{\infty }^{S_{t}}=1\right)$ when $\frac{P_{t}}{\alpha }<K_{t}$. □

The following Corollary is introduced without its proof, although, the arguments would be similar to Corollary 2.

**Corollary 3. ***It holds that:*

i) $SK_{\infty }^{K_{t}}\to \frac{P_{t}}{\alpha }$ as *S*_*t *_→ *∞ *and $\left(R_{\infty }^{K_{t}}\to 1\right)$ as *S*_*t *_→ *∞*

ii) $\left(SK_{\infty }^{K_{t}}\to 0\right)$ as *α *→ *∞ *and $\left(R_{\infty }^{K_{t}}\to 1\right)$ as *α *→ *∞*

iii) *If *$\frac{P_{t}}{\alpha }<\frac{S_{t}}{\alpha +1}$, then $SK_{\infty }^{K_{t}}\to \frac{P_{t}}{\alpha }$ as *λ*_1 _→ *∞ *and $\left(R_{\infty }^{K_{t}}\to 1\right)$ as *λ*_1 _→ *∞*. If $\frac{P_{t}}{\alpha }>\frac{S_{t}}{\alpha +1}$, then $SK_{\infty }^{K_{t}}\to \frac{S_{t}}{\alpha +1}$ as *λ*_1 _→ *∞ *and $R_{\infty }^{K_{t}}\to \frac{\alpha }{\alpha +1}$ as *λ*_1 _→ *∞*.

#### Hill numbers

The Hill number is defined as

$$n_{H}=\frac{\log(81)}{\log(K_{t}^{90}/K_{t}^{10})},$$

where $\left(K_{t}^{90}/K_{t}^{10}\right)$ is the response coefficient [6]. Solving for *SK* from (26) and by condition (24) we obtain

(30) $$\;\;\;\;\;\;[\text{SK}]=\frac{M_{1}+P_{t}+RS_{t}-\sqrt{{\left(M_{1}+P_{t}+RS_{t}\right)}^{2}-4RS_{t}P_{t}}}{2\alpha }.$$

We also find *K*_*t *_from (22) to be

(31) $$K_{t}=\frac{{\left[\text{SK}\right]}^{3}+v_{1}{\left[\text{SK}\right]}^{2}+v_{2}[\text{SK}]}{{\left[\text{SK}\right]}^{2}-u_{1}[\text{SK}]-u_{2}}.$$

Given $\left(R_{\infty }^{K_{t}}\right)$, we have $\left(R^{90}=0.9R_{\infty }^{K_{t}}\right)$ and $\left(R^{10}=0.1R_{\infty }^{K_{t}}\right)$. Then we can obtain *SK*^90 ^and *SK*^10 ^from (30) and finally, $\left(K_{t}^{90}\right)$ and $\left(K_{t}^{10}\right)$ from (31). This gives the Hill number *n*_*H*_.

### Steady-state approximation using tQSSA

The tQSSA replaces the free substrate *S*_*p *_as the slow variable by the total intact substrate concentration *S*_*p*_+ *S*_*p*_*P*, while retaining the quasi-steady-state assumption for the intermediate complexes. Since the extended model has 3 products (*S*_*p*_,*S* and *PK*), we introduce 2 slow variables (total *S*_*p *_and total *PK* concentrations).

Denote *C*_1 _= [*S*_*p*_*P*],*C*_2 _= [*S*_*p*_*PK*],*C*_3 _= [*SK*],*C*_4 _= [*PK*] and let *X *= *C*_2 _+ *C*_4 _to be the total *PK* concentration and *A *= [*S*_*p*_] + *C*_1 _to be the total *S*_*p *_concentration, respectively. Then it holds that

$$\begin{array}{l} P_{t}=[P]+C_{1}+X,\;K_{t}=[K]+C_{3}+X,\\ S_{t}=A+[S]+C_{2}+C_{3}. \end{array}$$

The variables *A* and *X* satisfy the following equations

(32) $$\frac{\text{dX}}{\text{dt}}=\kappa_{1}(P_{t}-C_{1}-X)(K_{t}-C_{3}-X)-\kappa_{-1}(X-C_{2}),$$

(33) $$\frac{\text{dA}}{\text{dt}}=-k_{1}C_{1}+k_{2}C_{3}-k_{3}C_{2}.$$

We hypothesize that the intermediate complexes *C*_1_,*C*_2_,*C*_3 _have faster dynamics then the active protein *A*. At steady-state, the intermediate complexes satisfy

(34) $$M_{1}C_{1}=(A-C_{1})(P_{t}-C_{1}-X),$$

(35) $$M_{3}C_{2}=(A-C_{1})(X-C_{2}),$$

(36) $$M_{2}C_{3}=(S_{t}-A-C_{2}-C_{3})(K_{t}-C_{3}-X).$$

The simulations of the tQSSA approach are shown in Figure 5.

## Competing interests

The authors declare that they have no competing interests.

## Authors' contributions

VS conceived the study and BSz performed the research. BSz and VS analyzed results and wrote the paper. Both authors read and approved the final manuscript.
